# Severe Multisystem Inflammatory Syndrome Associated With SARS‐CoV‐2 in a 31-Year-Old Male Patient: The First Clinical Case Report From the Republic of Cyprus

**DOI:** 10.7759/cureus.22640

**Published:** 2022-02-26

**Authors:** Despina Markoulaki, Stelios Iordanou, Demetris Koukios, Ioanna Christoldoulou, Panos Papadopoulos, Chrystalla Timiliotou-Matsentidou

**Affiliations:** 1 Intensive Care Unit, Limassol General Hospital, Limassol, CYP

**Keywords:** intensive care unit, acute respiratory distress syndrome [ards], sars-cov-2 (severe acute respiratory syndrome coronavirus -2), covid-19 mis-c, multisystem inflammatory syndrome in adults [mis-a]

## Abstract

Multisystem inflammatory syndrome (MIS) in adults associated with severe acute respiratory syndrome coronavirus 2 (SARS‐CoV‐2) infection is increasingly reported in published literature, although published reports remain sparse. In this report, we describe our first experience with a 31-year-old Caucasian male who developed severe MIS 31 days after a mild SARS‐CoV‐2 infection. The patient developed fever, elevated C-reactive protein (CRP), procalcitonin (PCT), reduced ejection fraction (EF), and shock. After extensive diagnostic work-up, nothing was found to justify his shock manifestation. A similar treatment to MIS in children (MIS-C) with immunoglobulins, corticosteroids, and anticoagulants led to a remarkable clinical improvement.

MIS in adults (MIS-A) can be fatal. The early identification of MIS plays a crucial role in the prompt initiation of suitable treatment. Therefore, differential diagnosis and exclusion of other causes of illness are of priority. We believe that MIS in children treatment guidelines can be reformed in a way to include MIS in adults as well.

## Introduction

An increasing number of studies in published literature reports multisystem inflammatory syndrome (MIS), in children (MIS-C), associated with severe acute respiratory syndrome coronavirus 2 (SARS‐CoV‐2) infections. Reports from Europe and North America have described instances with symptoms resembling Kawasaki syndrome, toxic shock, and MIS manifestations [1] requiring admission to intensive care units (ICUs). Treatment included mainly immunoglobulin and steroids [1]. At the same time, there has been an increasing number of reports in young adults [2] similar to that initially described in children [3,4].

Before October 2021, the case definition for MIS in adults (MIS-A) was not available. According to the recently published CDC (Centers for Disease Control) definition for MIS-A, the patient must be over 21 years of age, hospitalized for ≥24 hours, or with an illness resulting in death, which meets specific clinical and laboratory criteria, and in the absence of alternative diagnosis for the illness such as bacterial sepsis or exacerbation of a chronic medical condition [5]. Despite the recently published MIS-A definition, treatment recommendations are not yet available; therefore, in the majority of reports, the patients were treated according to the MIS-C recommendations.

From December 31, 2019, to late November 2021, 129,158 cases of SARS‐CoV‐2 have been reported in the Republic of Cyprus [6], including 589 deaths. To our knowledge, this is the first case in the Republic of Cyprus involving MIS associated with SARS‐CoV‐2 in an adult patient similar to that described in children [7].

## Case presentation

The case refers to a 31-year-old Caucasian male with a COVID-19 infection confirmed by reverse transcription-polymerase chain reaction (RT-PCR) of a specimen collected with a nasopharyngeal swab on February 12, 2021. His initial symptoms included mild fever and malaise. After an uneventful initial recovery, 31 days after his initial positive PCR test, the patient developed a sudden high fever, malaise, and difficulty breathing, which led him to seek medical attention.

The patient visited a private hospital with tachycardia of 140 beats per minute, pyrexia of 39.6°C, oxygen saturation of 90% (on ambient air), and a blood pressure of 70 over 45 mmHg. He was given 2 g ceftriaxone IV and then was immediately transferred to the Limassol General Hospital, Limassol, Cyprus, where he was admitted to the ICU.

**Figure 1:**
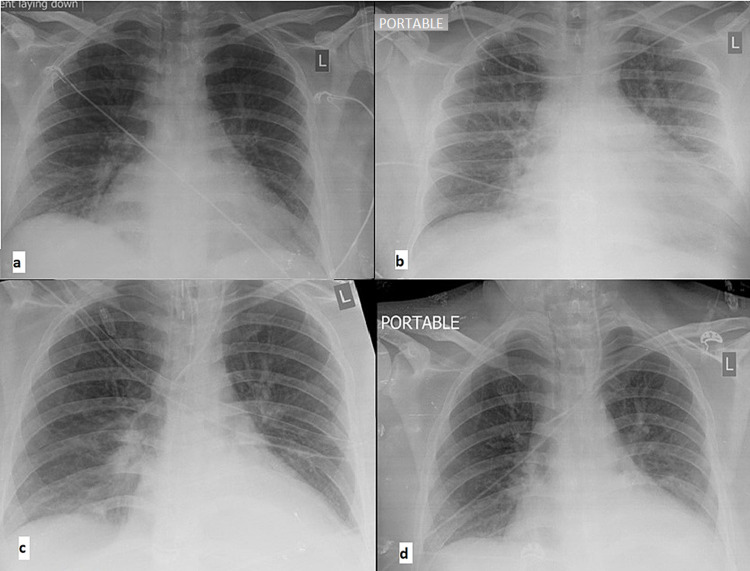
Chest x-rays (a) Admission day, (b) day two, (c) day five, and (d) day eight.

On admission, the patient was fully oriented but restless. He exhibited tachypnoea (30-45 RR per min). On a non-rebreathing mask at 15 litO_2_/min, arterial blood gases revealed a PO_2_ of 62 mmHg, PCO_2_ of 32 mmHg, pH of 7.39, and lactate levels less than 2 mmol/L. His chest x-rays were unremarkable in terms of infiltrates (Figure 1). Blood pressure on admission was 80/40 (mean arterial pressure [MAP]: 58) mmHg. Given this hemodynamic profile, vasopressor (noradrenaline) support was required and continued for several days. The maximum dose of noradrenaline was used on days two and three (0.1 mcg/kg/min) and stopped on day seven.

**Figure 2:**
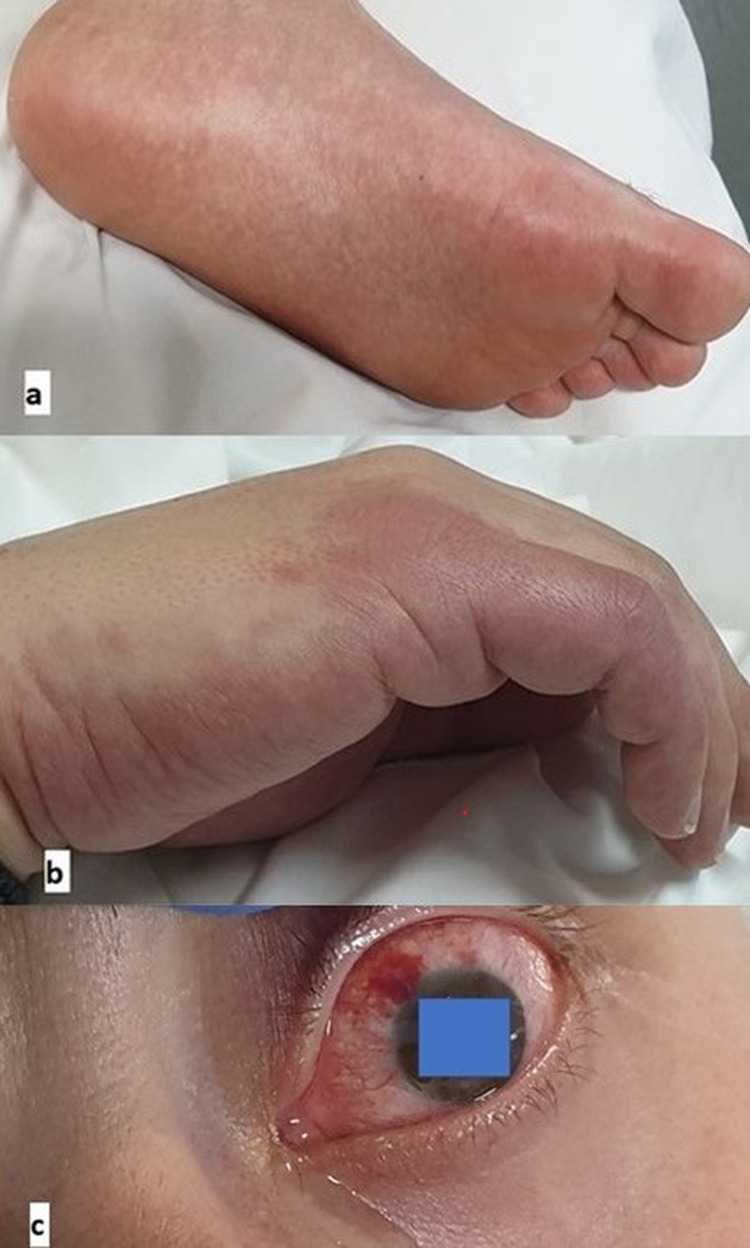
(a-c) Conjunctivitis and diffuse rash on palms and lower extremities on admission day

Physical examination revealed conjunctivitis, lip cheilitis, and diffuse rash on his palms and lower extremities (Figure 2).

The results of laboratory tests upon admission showed an elevated C-reactive protein (CRP) of 372.46 mg/L (<5), a lymphocyte count of 0.78 x 10^3^/μL (1-3.4), white blood cells (WBC) of 14.6 x 10^3^/μL (3.6-9.2), a troponin of 0.75 ng/mL (0 and 0.04), creatinine of 2.44 mg/dL (0.67-1.17), urea of 111 mg/dL (17-43), and procalcitonin (PCT) of 10 µg/L (<2) (Table 1). Specimens were collected according to the European Centre for Disease Prevention and Control (ECDC) [8] guidance and included a nasopharyngeal swab for SARS‐CoV‐2. The patient had no previous medical history and no known comorbidities. His ECG was unremarkable, and a transthoracic echocardiogram displayed a mild to moderately reduced ejection fraction (EF) of 50%-55% with a trace of pericardial effusion. A full-body contrast-enhanced CT revealed cervical and mesothorax lymphadenopathy and bronchiectasis without infiltrates.

**Table 1:** Laboratory results WBC: White blood cells; NEUT: Neutrophils; LYM: Lymphocytes; MONO: Monocytes; EOS: Eosinophils; BASO: Basophils; IG: Immature granulocytes; RBC: Red blood cells; HGB: Hemoglobin; HCT: Hematocrit; MCV: Mean corpuscular volume; MCH: Mean cell hemoglobin; PLT: Platelets; MPV: Mean platelet volume; LDH: Lactate dehydrogenase; CRP: C-reactive protein; PCT: Procalcitonin.

Laboratory	Day one	Day two	Day three	Day four	Day five	Day six	Day seven	Day eight	Day nine
WBC 10^3^/μL	14.62	22.11	22.62	17.25	7.66	4.61	6.55	5.16	8.84
NEUT%	91.8	95.2	90.2	89.6	86.3	83.1	86.5	84.7	89.2
LYM%	5.3	3.1	4.8	6.8	9.3	11.5	6.9	8.7	6.9
MONO%	1.5	1.2	2.7	2	3.3	4.3	5.3	5.4	2.4
EOS%	1	0.1	0.4	0.1	0	0	0	0	0.6
BASO%	0.1	0.2	0.2	0.2	0.1	0	0	0	0.1
IG%	0.30	0.20	1.7	1.3	1	1.10	1.10	1.2	0.8
NEUT 10^3^/μL	13.42	21.03	20.43	15.46	6.61	3.83	5.67	4.37	7.89
LYM 10^3^/μL	0.78	0.69	1.08	1.17	0.71	0.53	0.45	0.45	0.61
MONO 10^3^/μL	0.22	0.26	0.6	0.34	0.25	0.2	0.35	0.28	0.21
EOS 10^3^/μL	0.14	0.03	0.08	0.02	0	0	0	0	0.05
BASO 10^3^/μL	0.01	0.05	0.05	0.04	0.01	0	0.01	0	0.01
IG 10^3^/μL	0.05	0.05	0.38	0.22	0.08	0.05	0.07	0.06	0.07
RBC 10^3^/μL	4.62	44.4	4.33	4.18	3.74	3.38	3.52	3.61	3.85
HGB g/dL	12.8	12.6	12.3	11.6	10.5	9.6	9.8	10	10.9
HCT%	39.6	38	37.5	36.0	32.4	30.2	31.1	31.7	33.5
MCV fL	85.7	85.6	86.6	86.1	86.6	89.3	88.4	87.8	87
MCH pg	27.7	28.4	26.4	27.8	28.1	28.4	27.8	27.7	28.3
PLT 10^3^/μL	136	172	157	199	175	163	173	156	177
MPV fL	11.3	11.5	11.9	11.7	11.8	11.3	11.3	11	11
CRP mg/L	372.46	423.9	350.1	230	121	73.36	47	31.8	22.13
PCT ng/m		>10					2		
LDH	445	453	615	613	473	465	513	498	
Urea mg/dL	111	73	66	71	106	111	95	86	86
Creatinine	2.44	1.65	1.77	1.86	1.6	1.24	0.96	0.63	0.63
Ferritin ng/ml		3420		8060	6285	5503		3563	2815
Troponin	0.75	0.87		0.08	0.08				

On day 2, the patient turned into a prone position with no result in oxygenation (PO_2_/FiO_2_ ratio of 68 on non-rebreather mask [NRM] 100%/15 litO_2_). The patient was intubated and mechanically ventilated due to severe persistent tachypnoea and hypoxia (PO_2_/FiO_2_ ratio of 114 on FiO_2_ 1.00). That day’s laboratory tests revealed ferritin of 3420 ng/ml (peak on day 4: 8060 ng/ml) and an elevation of WBC to 22.11 x 10^3^/μL (peak on day 3: 22.62 x 10^3^/μL) (Tables 1, 2).

**Table 2:** Vasopressor infusion and dose, oxygen administration and method, PO2/FiO2 ratio and vital signs PO_2_: Partial pressure of oxygen; FiO_2_: Fraction of inspired oxygen; PEEP: Positive end-expiratory pressure.

	Day one	Day two		Day three	Day four	Day five	Day six	Day seven	Day eight	Day nine
Vital signs		Before intubation (prone position)	After intubation	After intubation					Extubation	
Temp (°C)	38.9	40		39.8	39.8	40	39.2	38.2	37.1	36.5
PO_2_/FiO_2_ ratio	62.7	68	114	100	100	136	130	200	340	>400
Oxygen administration and method	NRM 100%/15 litO_2_	NRM 100%/15 litO_2_	FiO_2_ 1.00	FiO_2 _0.65	FiO_2_ 0.70	FiO_2_ 0.55	FiO_2_ 0.45	Venti mask 60%/10 litO_2_	Nasal cannula 6 litO_2_	Nasal cannula 6 litO_2_
Respiratory rate, /min	49	36	20	20	18	20	10	16	14	18
PEEP, mmH_2_O	-	-	10	10	10	10	13	7	-	-
Total volume, ml	-	-	500-550	500-600	500-670	527-660	400-600	527-660	-	-
Noradrenaline infusion and dose mcg/kg/min	0.075	0.1	0.1	0.07	0.025	0.015	(Only for a few hours) 0.015		-	-
Kidney function, ml/kg/hour	1.84	0.98		2.72	2.57	2.21	2.08	2.53	1	1.45

All microbial testing was negative, as was a PCR for COVID-19. No bacteria were isolated in the bronchoalveolar lavage (BAL) specimens. Given that the patient's symptoms were similar to MIS associated with COVID-19, the patient was started on intravenous immunoglobulin (IVIG) 0.4 g/kg/day, methylprednisolone 62.5 mg three times daily, low molecular heparin 0.6 IU, and aspirin 75 mg daily (Table 3). Despite the negative sepsis screen, we continued the antimicrobial until discharge as initial PCT was elevated.

**Table 3:** Pharmacological treatment

	Day one	Day two	Day three	Day four	Day five	Day six	Day seven	Day eight	Day nine
Immunoglobulin 0.4 g/kg/day			0.4 g/kg/day	0.4 g/kg/day	0.4 g/kg/day	0.4 g/kg/day	0.4 g/kg/day	0.4 g/kg/day	
Methylprednisolone			62.5 mg x 3	62.5 mg x 3	62.5 mg x 3	62.5 mg x 3	62.5 mg x 3	62.5 mg x 3	62.5 mg x 3
Enoxaparin			6000 IU x 1	6000 IU x 1	6000 IU x 1	4000 IU x 2	4000 IU x 2	4000 IU x 2	4000 IU x 2
Aspirin			75 mg x 1	75 mg x 1	75 mg x 1	75 mg x 1	75 mg x 1	75 mg x 1	75 mg x 1

Outcome

**Figure 3:**
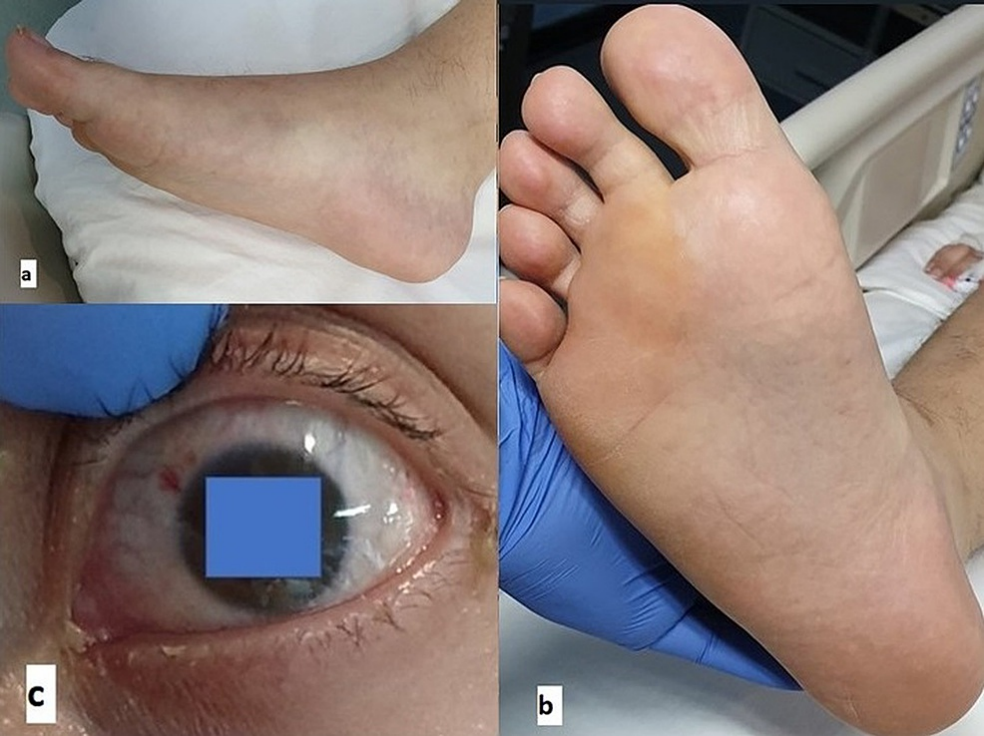
(a-c) After the treatment was provided, conjunctivitis and diffuse rash on palms and lower extremities disappeared on day five

**Figure 4:**
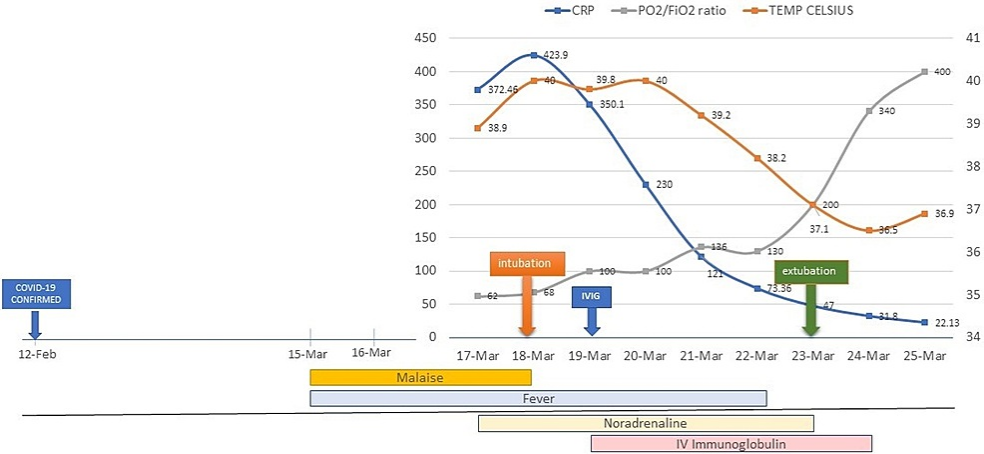
Patient timeline progress

The patient's condition improved with the treatment provided. He remained on mechanical ventilation for six days, and oxygenation returned to near normal by the seventh day. The exanthem disappeared on the fifth day, and the fever settled on the sixth day (Figures 3, 4). Repeat transthoracic echocardiogram (ECHO) on the ninth day showed an improvement to 65%. Noradrenaline was discontinued on the eighth day. The patient was discharged from the ICU fully recovered on day nine.

## Discussion

MIS has been linked with COVID-19. It is likely underdiagnosed, and the pathogenesis remains undefined [2], but there is evidence that its increasing post-COVID-19 occurrence probably shows an association with the dysregulation of the immune system or an antibody-mediated process due to the infection [9,10]. MIS can also occur after an asymptomatic COVID-19 infection in children and adults [11,12]. Although this can result in severe illness, most adult patients survive [10,11].

Bastug et al. illustrated that hypotension, tachycardia, fever, conjunctivitis, diffuse exanthem, dyspnea, and cough are among the most prevalent MIS clinical presentation as well as gastrointestinal symptoms and reduced left ventricular EF. More than 40% of the patients may require vasopressors for hypotension, and almost 20% may require mechanical ventilation [12]. A recent study [10] found that 98% (207 out of 211) of the cases reporting MIS-A had a current or past history of COVID-19 infection, 51% (110 of 214) presented with increased severity of illness requiring vasoactive medications, and 57% were admitted to ICU. Among all patients, 7% died.

The recently published CDC case definition criteria for MIS-A [5] is a step forward for a better understanding of the syndrome and has the potential of encouraging clinicians toward MIS-A reporting, in order to increase awareness regarding the syndrome and encourage information sharing. At the time of our patient ICU admission, the case definition for MIS-A was not yet available. Therefore, the identification relied on similar reports found in the published literature [3,4,12,13].

According to recently published criteria on the syndrome [5], the patient's symptom manifestations, and medical history, we believe our 31-year-old patient met all of the criteria of diagnosis. He presented with fever (≥38.0°C) for ≥24 hours before hospitalization, reduced EF, rash and non-purulent conjunctivitis, and shock, which are not attributable to other causes or medical therapy, elevated CRP and PCT levels, a recent history of COVID-19 infection documented by RT-PCR, and no alternative diagnosis for the illness.

He was characterized as a severe case of MIS-A since respiratory and circulatory support was needed. Management of the patient was guided by the available reports on MIS-A, similar to what has been reported for MIS-C [3,4,12,13]. His clinical condition improved remarkably after the treatment. Although MIS-A associated with COVID-19 is increasingly being reported [4,12], there are no optimal treatment guidelines, and patients in most of the cases are treated similar to the treatment options used in children [14]. However, effective surveillance can provide a better understanding of MIS-A manifestations and potentially can identify more effective therapies or strengthen the current treatment protocols.

## Conclusions

Healthy young adult patients may present with severe multiorgan dysfunction several weeks after a mild infection with SARS-Cov2 requiring respiratory and cardiac ICU support. Since early MIS-A identification plays a significant role in prompt pharmacological treatment initiation and patient recovery, rapid exclusion of other causes is of great importance.

MIS-A identification relies mainly on clinical symptoms; therefore, clinicians should consider MIS-A as a possible cause for patients with SARS-Cov2 infection history. Local authorities should promote awareness and reporting. Treatment guideline for MIS-A is currently not available; but given the rapid response in our patient, we believe that the MIS-C treatment guideline can be adapted for MIS-A.
